# Pectins, Endopolygalacturonases, and Bioenergy

**DOI:** 10.3389/fpls.2016.01401

**Published:** 2016-09-20

**Authors:** Mariana B. G. Latarullo, Eveline Q. P. Tavares, Gabriel Padilla, Débora C. C. Leite, Marcos S. Buckeridge

**Affiliations:** ^1^Laboratory of Plant Physiological Ecology, Department of Botany, Institute of Biosciences, University of São Paulo São Paulo, Brazil; ^2^Bioproducts Laboratory, Department of Microbiology, Institute of Biomedical Sciences, University of São Paulo São Paulo, Brazil

**Keywords:** bioethanol, pectinase, endopolygalacturonase, grasses, cell wall, bioenergy, ethanol

## Abstract

The precise disassembly of the extracellular matrix of some plant species used as feedstocks for bioenergy production continues to be a major barrier to reach reasonable cost effective bioethanol production. One solution has been the use of pretreatments, which can be effective, but increase even more the cost of processing and also lead to loss of cell wall materials that could otherwise be used in industry. Although pectins are known to account for a relatively low proportion of walls of grasses, their role in recalcitrance to hydrolysis has been shown to be important. In this mini-review, we examine the importance of pectins for cell wall hydrolysis highlighting the work associated with bioenergy. Here we focus on the importance of endopolygalacturonases (EPGs) discovered to date. The EPGs cataloged by CAZy were screened, revealing that most sequences, as well as the scarce structural work performed with EPGs, are from fungi (mostly *Aspergillus niger*). The comparisons among the EPG from different microorganisms, suggests that EPGs from bacteria and grasses display higher similarity than each of them with fungi. This compilation strongly suggests that structural and functional studies of EPGs, mainly from plants and bacteria, should be a priority of research regarding the use of pectinases for bioenergy production purposes.

## Introduction

Bioenergy can be produced from several species and in different ways. The basic idea is to transform hydrocarbons produced by plants and algae –mostly in the form of sugars and lipids– into compounds similar to gasoline, diesel, and ethanol. In the latter case, there are only a handful of plant species that have been considered or effectively used for bioenergy production in large scale. Among these there are the ones that produce high concentrations of sucrose (e.g., sugarcane and sugar beet), starch (e.g., maize), lignocellulose from woody materials (e.g., poplar and willow) or grasses (e.g., corn stove, switchgrass, miscanthus, and sugarcane) (De Souza et al., 2013a). Other residues, such as apple pomace, citrus, and sugar beet, which are rich in pectins have been suggested as possible sources of carbon for bioethanol production (Edwards and Doran-Peterson, 2012).

Maize (*Zea mays*) and sugarcane (*Saccharum* sp.) are the main feedstocks used in large scale for bioethanol generation, with the USA and Brazil being the world’s largest producers (RFA, 2015). The bioethanol from sugarcane can be produced at lower costs in comparison to maize (Méjean and Hope, 2010) probably due to the energy balance in the production process which has been optimized since the early 20th century (De Souza et al., 2013a; Loqué et al., 2015).

**FIGURE 1:**
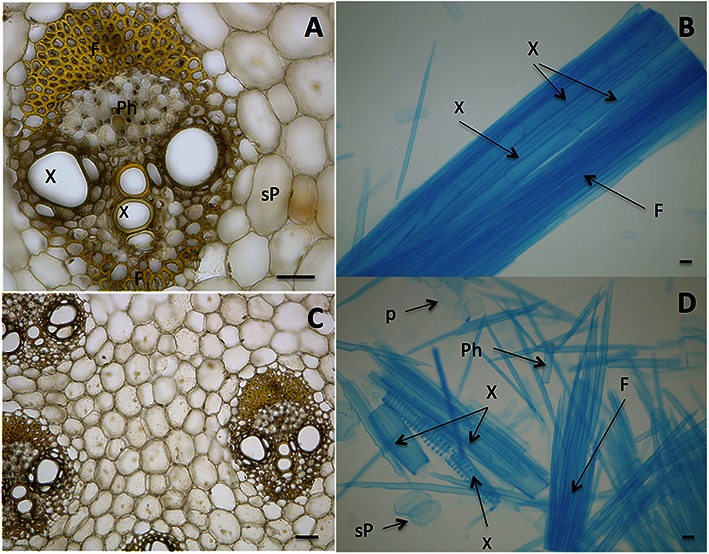
**Biomass units of sugarcane stem (culm) with cells bound by middle lamellae. (A,C)** Stele, the vascular bundle showing Fibers (F), Phloem (Ph), Xylem (X), sucrose-storage parenchyma (sP). **(B)** Macerated tissue showing a detail of an isolated stele that still maintains adhesion among cells. **(D)** Cells of sugarcane stem without the binding of middle lamellae. Cells are named as in **(A)**. The cell separation was prepared according to Franklin (1945) and cells were stained with toluidine blue **(B,D)** and iodine-potassium iodide **(A,C)**. Bars represent 100 μm.

The first generation bioethanol (1G) consists in fermenting sucrose from the juice. Sucrose is extracted from large vacuoles present in cells of the stem (**Figures 1A,C**). Sucrose is then used in fermentation tanks to produce ethanol using *Saccharomyces cerevisiae* (Amorim et al., 2011). Although this process became extremely efficient along decades of engineering development, some of the sucrose is still lost during the process due to difficulty to open all sucrose-storage cells of the culm. These cells cannot all be broken probably due to the strength of the cell walls as well as their junctions (the middle lamellae), so that groups of cells can resist to the mechanical forces involved in the milling and hot water extraction during the 1G industrial process (**Figures 1B,D**).

Second Generation (2G) bioethanol production consists in using different strategies to extract the sugars present in the cell walls. For this purpose, some suggested feedstocks are woody materials (e.g., poplar, willow, eucalyptus), grass stems, that contain proportionally more cell walls than sucrose (e.g., miscanthus and switchgrass) and also from plant residues, such as corn stover, wheat straw, sugarcane bagasse, sugar beet, citrus, and apple residues.

The 2G processes have been designed to include biomass pretreatment (Soccol et al., 2010; Pu et al., 2013; Martins et al., 2015; Pereira et al., 2015) aimed at granting access to cellulose. Pretreatments are known to cause losses of hemicelluloses and pectins (DeMartini et al., 2013; Pu et al., 2013), decreasing yield.

The raw material employed in 2G technologies is the plant cell wall, a complex conglomerate of three polysaccharide domains (pectins, hemicelluloses, and cellulose) which are cross-linked with lignin (Buckeridge et al., 2015). This Mini-review aims to look on the role of pectins on cell wall recalcitrance to hydrolysis and the potential of pectinases for bioenergy purposes. We revised the literature about the use of pectinases to understand cell wall hydrolysis and show that research made on these enzymes is quite limited since only a few have been purified and characterized. Here we focus on endopolygalacturonases, which are the most well known among the pectinases. Increasing knowledge about the structural and functional aspects of these enzymes might bring new possibilities for their use in biomass hydrolysis and bioethanol production.

## Cell Walls, Pectins, and Bioenergy

Cell walls are composites made of polymers with diverse structures that are attached by covalent and non-covalent linkages, forming an extremely complex network that constitutes the Glycomic Code (Buckeridge and de Souza, 2014; Buckeridge et al., 2015; Tavares and Buckeridge, 2015)

Among the grasses used for bioenergy purposes, maize (Carpita, 1984), sugarcane (De Souza et al., 2013b), and miscanthus (De Souza et al., 2015) are among the most profoundly studied species. The architecture of the walls of grasses is named Type II, which has arabinoxylans as the main hemicellulose and relatively low proportions of pectins (ca. 2–10% of the wall in sugarcane and miscanthus) (Mohnen, 2008; Vogel, 2008; De Souza et al., 2013b, 2015). Maize and sugarcane are known to form “packages” of cellulose *micro*fibrils (*macro*fibrils) bound to xyloglucan and xylans, whereas pectins seem to form a separate domain with more soluble polysaccharides into which the other cell wall domains are embedded (Ding et al., 2012; Buckeridge et al., 2015). Despite its low content in grasses, pectins are possibly involved in bindings with lignin in sugarcane and miscanthus, conferring recalcitrance to hydrolysis (De Souza et al., 2013b, 2015).

It is also widely known that pectins are the main components of the middle lamella. Thus, besides hampering wall hydrolysis and thus preventing efficient release of sugars for 2G bioethanol, the presence of pectins in the middle lamella can also be a barrier to the release of cells from tissues in 1G processes, interfering in sucrose extraction (De Souza et al., 2013b, 2015).

The hydrolysis step required for disassembly of cell walls and their polymers is regarded as one of the major bottlenecks for 2G ethanol production. Thus, understanding the mechanisms of breakdown of the cell walls will help to determine the most efficient strategies to reduce costs of production (Turumtay, 2015). Considering what is known about the structure and fine structure of the pectic and hemicellulosic polymers in the walls of sugarcane and miscanthus, the degradation of the cell walls of sugarcane, for instance, is thought to require at least 18 different types of enzymes, several of them related to pectin attack (De Souza et al., 2013b).

Pectins are composed mainly of three polysaccharides, including homogalacturonan present within the middle lamella, which can be acetylated and/or methylated. Rhamnogalacturonans I and II present a more complex structure, composed of a main chain of galacturonic acid interspaced with residues of rhamnose which are branched with chains of arabinans, galactans, and arabinogalactans.

Usually referred to as accessory enzymes, the main required enzymes to hydrolyze pectins are endopolygalacturonases (EPGs), acetyl and methylesterases, α-arabinofuranosidases and β-galactosidases. The fact that some linkages between carbohydrates and phenylpropanoids would have to be broken in order to allow attack to pectin, possibly more enzymes than what is currently known might be involved in the complete hydrolysis of this polysaccharide (Rytioja et al., 2014).

Experiments have been performed with pectin-rich materials such as citrus waste (Edwards and Doran-Peterson, 2012; Awan et al., 2013). Employing this biomass, a mixture of *S. cerevisiae* and *Candida parapsilosis* have been successfully fermented. Also, the use of enzymes from *Xanthomonas axonopodis* (a Gram negative pathogen that confers canker to citrus) led to high efficiency in ethanol production.

The effort on prospecting pectinases has been mostly on fungal enzymes whereas some plant enzymes have also been studied in systems of agronomic importance. Conversely, in plants, the attack upon the pectins of cell wall and middle lamella have been largely described for abscission zone formation (González-Carranza et al., 2002), fruit ripening (Prasanna et al., 2007) and aerenchyma formation (Gunawardena et al., 2001). As a result, relatively little is known about the structural and functional diversity of pectinases in plant and microbe metabolisms.

The roles of pectin in biomass yield and processing for bioenergy have been recently reviewed (Xiao and Anderson, 2013). Since pectins represent the class of polysaccharides responsible for adhesion to neighbor cells, it could be decreased due to pectinase activity. Another important role is the control of penetration of other enzymes into the biomass network, since pectins are thought to be responsible for determination of cell wall porosity (Baron-Epel et al., 1988), limiting the size and dimensions of enzymes allowed to penetrate the wall (Buckeridge et al., 2015). A search in the CAZy databank gave 210 sequences of EPGs (GH28 family, EPG 3.2.1.15), 46 pectin acetyl esterases (CE12 and CE13 families, PAE 3.1.1.) and 44 pectin methyl esterases (CE8 family, PME 3.1.1.11) (Lombard et al., 2014). Thus, EPGs are by far the best-known enzyme family associated to the homogalacturonan hydrolysis.

## Potencial Use of Pectin and Pectinases for Bioenergy

Depolymerases such as polygalacturonases are classified as endo- or exopolygalacturonases according to their mode of action. Exopolygalacturonases attack the non-reducing end of the polymer, generating the monosaccharide galacturonic acid. EPGs hydrolyze inner linkages within homogalacturonan molecules, originating oligogalacturonides of different sizes.

Endopolygalacturonases play important roles in fruit ripening. Smith et al. (1990) found that down-regulation of EPG expression led to a reduction in enzyme activity between 5 and 50%, increasing ripe fruit shelf life. Similar results were observed for apple and strawberry fruits. Moreover, microscopic analysis of transgenic fruits revealed smaller inter-cellular spaces and higher cellular adhesion (Atkinson et al., 2012; Posé et al., 2013).

Because the proportion of pectins in grasses is rather low, studies are scarce about their effect on saccharification. Some evidence from experiments with dicot plants produced promising results. The addition of pectinases to enzyme cocktails can increase biomass hydrolysis in *Arabidopsis* (Lionetti et al., 2010). Supplementing the commercial enzyme cocktail Celluclast 1.5 L (which is mainly composed of cellulases) with fungal EPG or overexpression of endogenous pectin methyl esterases, increased the efficiency of enzymatic hydrolysis of cell walls. It is not yet known by which mechanisms this process takes place, but it is probable that these are related to the attack performed by pectinases in general, which results in the removal of pectins that prevent other enzymes to act. Thus, pectin connections in the cell wall matrix can affect saccharification and pretreatment, so that EPGs could eliminate the need for acid pretreatment, reducing costs and environmental impacts.

Concerning microbe EPGs, *Aspergillus niger* is by far the most studied fungi species and at least seven different types of the enzyme have been described and classified as EPG I, II, A, B, C, D, and E (Bussink et al., 1992), producing substrates with different degrees of polymerization (DP). EPGI produces large amounts of oligogalacturonides with DP 5, whereas EPG II products range from DP 6 to 15 (Cook et al., 1999). Moreover, they have distinct properties regarding optimal pH and temperature, substrate specificity and molecular weight. This wide variety of pectinases found in *A. niger* reflects the high complexity of cell wall architecture (i.e., the Glycomic Code) within and among cell walls of different plant species.

Currently, it is possible to find in the literature the characterization of different EPGs produced by fungi, bacteria, archaea, plants, insects, and nematodes. The most studied organisms are fungi and plants and only fungi EPGs had their 3D structure characterized (van Santen et al., 1999; Pouderoyen et al., 2003).

## The Diversity of EPGs in Microorganisms and Plants

In order to evaluate the diversity of characterized EPGs, all 210 sequences classified as such (3.2.1.15) were selected from the GH28 family (Lombard et al., 2014). From those, 177 protein sequences harboring predicted GH28 and pectin lyase domains were identified according to InterPro database (Marchler-Bauer et al., 2014). As a result, a total of 122 (68%), 39 (22%), 12 (0,8%), and 4 (0,2%) represented fungi, plant, bacteria, and other eukaryotes EPGs, respectively.

The most represented phylum among fungi was Ascomycota (70 sequences), mainly composed of *Aspergillus* species (21 sequences). Among the *Aspergilli*, those from *A. niger* were the most abundant (five sequences). Following Ascomycota, Oomycota was the second most represented fungi phylum, presenting nine sequences, all from *Phytophthora parasitica*. Concerning bacteria, the most common sub-order was Enterobacteriaceae, with two sequences from *Pectobacterium carotovorum*.

Plant sequences were from Solanaceae (10 sequences): nine from *Solanum lycopersicum* and one from *Nicotiana tabacum*. Among plant species, the families better represented within GH28 (Solanaceae, Rosaceae, Leguminosae) do not have large application in the biofuel industry. Although the grasses are more established as promising feedstocks for biofuel production (Somerville et al., 2010), according to CAZy, no grass endopolygalacturonase figure among the characterized GH28.

In order to compare similarities among plants, bacteria and fungi, the sequences from the above mentioned species (only the most represented from each mentioned fungi phylum, bacteria sub-order or plant family) were aligned using MUSCLE available from MEGA v. 7 (Kumar et al., 2016). Due to the role played by grasses as feedstocks for bioenergy, all GH28 grass EPGs were added to the alignment (20 sequences from *Z. mays*, one sequence from *Sorghum bicolor* and one from *Setaria italica*). Next, a phylogenetic tree derived from the alignment was constructed using Maximum Likelihood as a model and Tamura-Nei method (Tamura and Nei, 1993) with 1000 bootstrap replications.

**FIGURE 2:**
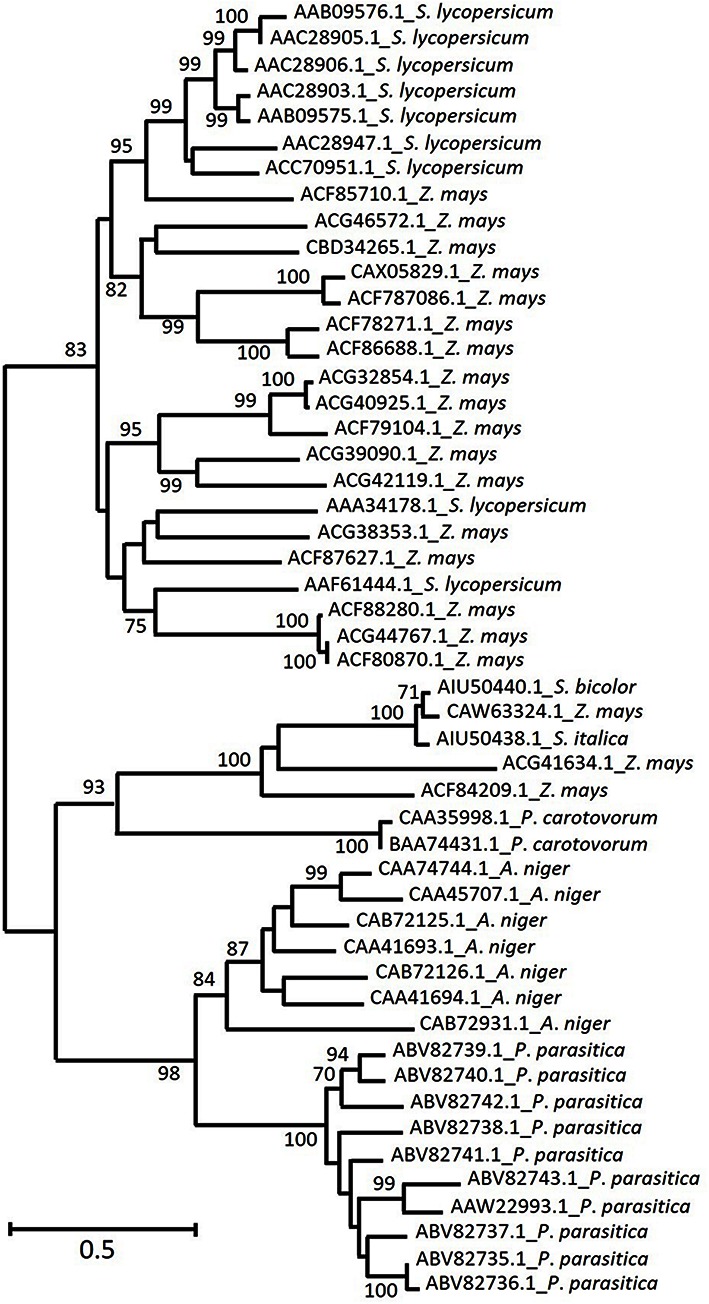
**Homology of deduced amino acid sequences from the most represented plant and microbe EPG among the characterized enzymes from CAZy GH28.** Predicted EPGs from grasses (*Setaria italica*, *Sorghum Bicolor*, and *Zea mays* – not yet characterized) were added to the alignment. The arabic numbers indicate bootstrap values and phylogenetic groups identified, respectively. The maximum likelihood tree was chosen accordingly to the highest ranked substitution model (Tajima-Nei) and 1000 bootstrap replications. NCBI GenBank numbers are indicated in each sequence.

The phylogenetic tree in **Figure 2** shows that plant EPG diversity appears to be higher than the one observed for fungi. Whereas microbes and grasses (including three out of 20 *Z. mays* sequences) EPG are clustered separately, the majority of *Z. mays* sequences are allocated in two major clades, one among the grass sequences and another among *S. lycopersicum* EPG sequences. *S. lycopersicum* EPGs play important roles during fruit ripening, germination and leaf and flower abscission. Although *S. lycopersicum* sequences divergence is not well supported by bootstrap (<70), they are described below as two different clusters in order to better organize the presentation of functional and transcriptional information.

Increased galacturonic acid levels along with increased accumulation of LeXPG1 mRNA, suggests that this enzyme (AAF61444.1) is required for radicle protrusion (Sitrit et al., 1999), whereas another tomato enzyme (AAA34178.1) has a fruit ripening-specific pattern (Bird et al., 1988). The majority of the remaining *S. lycopersicum* sequences (AAC28905.1, AAC28906.1, AAC28903.1, AAC28947.1, AAB09575.1, AAB09576.1) is related to abscission zone formation within leaves and flowers (Kalaitzis et al., 1997; Hong and Tucker, 1998). The specificity of these enzymes seems to be a relevant issue since divergent sequences are thought to play different biological roles (Bird et al., 1988), besides displaying different temporal expression patterns (Kalaitzis et al., 1997). It is hard to speculate which roles might be played by *Z. mays* sequences that were clustered together with *S. lycopersicum* ones. However, it is important to note that protein sequence identity between *Z. mays* (ACF85710.1) and *S. lycopersicum* range from 47 to 50%. This level of similarity is higher than the one observed among different *S. lycopersicum* EPG expressed on abscission zones compared to the ones expressed in fruit tissue (38–41%) (Kalaitzis et al., 1997). On the other hand, the identity between those related to leaf and flower abscission was much higher, 76–93%.

Fungi EPG characterization does not cover the same aspects addressed in plant EPG. Reports on functional characterization are available, concerning optimum pH, temperature and mode of action upon homogalacturonans with different degrees of polymerization. Although sugar beet pectins have been used to induce enzyme production, the activity of purified/heterologous EPG upon substrates derived from plants used as bioenergy feedstocks have not been reported. The change of substrates for yeast fermentation from monosaccharides (e.g., galacturonic acid or rhamnose) to polysaccharides (polygalacturonic acid) did not result in increased expression of *A. niger* EPG *pga*A and *pga*B and authors suggested that fungi metabolism was not promptly adapted to the new carbon source (Pařenicová et al., 2000a). The processivity of *A. niger pga*D was precisely and elegantly described upon lemon pectins (Pařenicová et al., 2000b) and polygalacturonan (Pařenicova et al., 1998). However, studies like these have not yet been performed using structurally well characterized polysaccharides from *Z. mays*, sugarcane or miscanthus (Carpita et al., 2001; De Souza et al., 2013b, 2015).

Interestingly, one clade exclusively composed of grass EPG is located separately from *S. lycopersicum* and from other *Z. mays* sequences, which might represent an interesting target for biotechnology research. These predicted proteins seem to be more closely related to microbial ones than to other proteins of plant species, although this divergence is not well supported by bootstrap values and might require further research in order to be confirmed.

Whereas the physiological roles of plant EPGs are reasonably well described, fungi EPG characterization does not cover the same functional aspects addressed in plants. Conversely, reports on plant EPG do not characterize target enzymes in respect of their activity upon a large range of substrates.

Evidently, all efforts toward enzyme prospection have a high value for bioenergy, providing new alternatives to create enzyme cocktails and complement the existing ones. Research on plant EPGs plays an important role in deciphering how they act during physiological processes in which the cell wall is modified and/or hydrolyzed. However, very few of these approaches fill the gap concerning how plant and microbe EPG act upon carbohydrates from bioenergy feedstocks.

## Concluding Remarks

Although in small proportion in walls of grasses and wood biomass feedstocks, pectins can play an important role to circumvent cell wall recalcitrance to hydrolysis.

The literature on EPG focusing on mechanisms of hydrolysis as well as the catalogs of related genes are quite limited, being restricted mainly to fungi (overall, *A. niger*) and a few species of bacteria and plants. The focus on comparisons among pectinases from different microorganisms, suggests that EPGs from bacteria and grasses display higher similarity than each of them with fungi. The fact that the structural and functional properties of EPGs are scarce suggests that such studies should be regarded as a priority in bioenergy research.

Due to the role of pectins on cell wall porosity and cell adhesion, hydrolysis of bioenergy feedstocks with EPG might aid significantly to decrease recalcitrance to hydrolysis, helping both 1G and 2G bioethanol production processes.

## Author Contributions

ML composed the first draft of the manuscript and worked with ET on the phylogeny. ET performed the study of the sequences and produced the phylogeny, with the help of ML under supervision of MB. DL produced **Figure 1**. GP and MB supervised the study. All authors worked on writing. MB and ET composed the final version and the main argument of the manuscript.

## Conflict of Interest Statement

The authors declare that the research was conducted in the absence of any commercial or financial relationships that could be construed as a potential conflict of interest.
