# Corrigendum: Pectins, Endopolygalacturonases, and Bioenergy

**DOI:** 10.3389/fpls.2017.02160

**Published:** 2017-12-14

**Authors:** Mariana B. G. Latarullo, Eveline Q. P. Tavares, Gabriel Padilla, Débora C. C. Leite, Marcos S. Buckeridge

**Affiliations:** ^1^Laboratory of Plant Physiological Ecology, Department of Botany, Institute of Biosciences, University of São Paulo, São Paulo, Brazil; ^2^Bioproducts Laboratory, Department of Microbiology, Institute of Biomedical Sciences, University of São Paulo, São Paulo, Brazil

**Keywords:** bioethanol, pectinase, endopolygalacturonase, grasses, cell wall, bioenergy, ethanol

An author name was incorrectly spelled as **Gabriel Padilla Maldonado**. The correct spelling is **Gabriel Padilla**. The authors apologize for this error and state that this does not change the scientific conclusions of the article in any way.

The original article has been updated.

## Conflict of interest statement

The authors declare that the research was conducted in the absence of any commercial or financial relationships that could be construed as a potential conflict of interest.

